# A Comprehensive Review: Molecular Diagnostics and Multi-Omics Approaches to Understanding Bovine Respiratory Disease

**DOI:** 10.3390/vetsci12111095

**Published:** 2025-11-17

**Authors:** Stephanie O’Donoghue, Sinéad M. Waters, Derek W. Morris, Bernadette Earley

**Affiliations:** 1 Animal and Bioscience Research Department, Animal and Grassland Research and Innovation Centre (AGRIC), Teagasc, Grange, Dunsany, C15 PW93 Co. Meath, Ireland; 2 School of Biological and Chemical Sciences, University of Galway, H91 TK33 Galway, Ireland

**Keywords:** bovine respiratory disease, diagnostics, microbiome, virome, next-generation sequencing, transcriptomics

## Abstract

Bovine respiratory disease (BRD) is a multifactorial syndrome and a leading cause of morbidity and economic loss in global cattle production. Next-generation sequencing (NGS) platforms, including Illumina and Oxford Nanopore Technologies (ONT), have enabled high-resolution profiling of the bovine respiratory microbiome and virome, revealing novel viral contributors such as bovine rhinitis A virus (BRAV) and influenza D virus (IDV). Transcriptomic approaches, including RNA sequencing (RNA-Seq) and microRNA (miRNA) profiling, provide insights into host immune responses and identify potential biomarkers for disease prediction. Traditional diagnostic methods—culture, ELISA, and immunohistochemistry—are increasingly complemented by PCR-based and metagenomic techniques, improving sensitivity and specificity. Despite technological progress, gaps remain in virome characterization, miRNA function, and the integration of multi-omics data. Standardized protocols and longitudinal studies are needed to validate microbial signatures and support field-deployable diagnostics. Advances in bioinformatics, particularly network-based integrative pipelines, are becoming essential for harmonizing multi-omics datasets and revealing complex host–pathogen interactions. The objective of this comprehensive review was to synthesize current understanding of the bovine transcriptomic response to BRD as well as the respiratory microbiome and virome, emphasizing how advanced sequencing technologies have transformed microbial profiling and molecular diagnostics in BRD.

## Simple Summary

Bovine Respiratory Disease (BRD) remains a major cattle health challenge, driven by viral, bacterial, and environmental factors. Integrated approaches using RNA-Seq and non-coding RNA profiling have revealed key host immune responses, while bacterial and viral metagenomics enable unbiased detection of known and emerging pathogens. Complementary respiratory microbiome studies highlight microbial dynamics influencing disease susceptibility and progression. Emerging technologies, including Oxford Nanopore sequencing, allow rapid species-level identification and real-time diagnostics. Combining host transcriptomics, viral metagenomics, and microbiome analyses provides a comprehensive understanding of BRD pathogenesis, supporting improved interventions, vaccines, and evidence-based disease management strategies in cattle.

## 1. Introduction

Bovine respiratory disease (BRD) is a complex, multifactorial syndrome and remains the leading cause of respiratory illness and mortality in cattle worldwide. It imposes significant economic burdens due to reduced productivity, increased veterinary costs, and animal losses [1,2,3]. The complex nature of BRD and the involvement of multiple pathogens and disease-causing factors can often complicate disease diagnosis. Traditionally, BRD diagnosis has been and remains centered on the observation of clinical signs of infection, as well as imaging techniques for the detection of lung lesions and certain behavioral parameters relating to infection [3]. Although a central part of BRD diagnostics, these methods can lack sensitivity in the case of subclinical disease and specificity regarding the pathogen causing the initial infection. In addition to these clinical-based approaches, lab-based approaches, such as pathogen culture, immunohistochemistry and enzyme linked immunosorbent assay (ELISA) have been widely adopted and have shown success in the diagnosis of BRD. However, these approaches are limited in their capabilities to identify novel pathogens and sensitivity. Furthermore, some BRD pathogens can be difficult to culture which can also lead to bias in results. The advancement in molecular methods such as PCR allowed for more advanced BRD diagnostics, with many studies employing this approach for the detection of BRD-associated pathogens [4,5]. However, PCR-based techniques often fall short in detecting unknown species.

Advances in molecular diagnostics have begun to address these limitations. Next-generation sequencing approaches have been widely used to study BRD pathogenesis and the immune response of cattle to both naturally acquired [6,7,8] and experimentally induced BRD [9,10,11]. These studies were able to identify genes differentially expressed across a range of different tissue types in response to BRD infection, as well as their associated biological pathways. These differentially expressed genes (DEGs) with could offer potential as biomarkers in future BRD screening approaches. In addition to host immunity, NGS approaches have also been used to investigate the respiratory microbiome during BRD infection. Central to BRD pathogenesis is the bovine respiratory microbiome—a diverse community of bacteria that supports mucosal homeostasis and modulates immune responses, is central to BRD pathogenesis. When this microbial balance is disrupted, a condition known as dysbiosis, cattle become more susceptible to colonization by pathogenic organisms and subsequent disease progression. The interplay between microbial communities, host immunity, and environmental stressors underpins the complexity of BRD [12,13]. For example, a study utilized NGS technologies to analyze lung and mediastinal lymph node microbiomes in both healthy and BRD-affected cattle, revealing significantly higher bacterial loads in diseased lungs [14] (Johnston et al. (2017)). Notably, they identified *Sneathia amnii* strain SN35—a previously unassociated member of the *Leptotrichiaceae* family—highlighting the potential of sequencing to uncover novel etiological agents [14].

In addition to bacterial communities, the bovine respiratory virome plays a critical yet underexplored role in BRD. This virome includes both pathogenic and commensal viruses that influence host–pathogen interactions and immune modulation. Virome composition is dynamic and influenced by factors such as stress, immune status, co-infections, and geographic location [13,15,16,17]. Metagenomic studies have expanded the known viral repertoire associated with BRD, showing that affected cattle often harbor viruses beyond classical pathogens such as BoHV-1, BPI-3, and BRSV. Emerging viruses like bovine rhinitis A virus (BRAV) and influenza D virus (IDV) have also been implicated [18]. Recent advances in high-throughput sequencing, particularly portable platforms like ONT MinION, have enabled rapid detection and genomic characterization of both established and emerging viruses, including BoCV, BRBV, BoNV, and UTPV1 [19,20,21].

Integrating metagenomic and viromic data through second- and third-generation sequencing technologies offers high-resolution insights into microbial interactions and their contributions to BRD pathophysiology. These approaches facilitate the detection of novel pathogens, quantification of microbial load, and identification of microbial signatures predictive of disease states. Moreover, portable sequencing devices like the MinION support point-of-care diagnostics and real-time surveillance, enhancing both research and field-based disease management. This comprehensive review synthesizes current understanding of the bovine transcriptomic response to BRD as well as the respiratory microbiome and virome, emphasizing how advanced sequencing technologies have transformed microbial profiling and molecular diagnostics in BRD. These innovations hold promise for improving disease detection, guiding vaccine development, and refining therapeutic strategies.

## 2. Materials and Methods

To provide a comprehensive overview of research progression in BRD, a structured literature search was conducted across PubMed, Scopus, Web of Science, and ScienceDirect, with supplementary searches in Google Scholar. No restrictions were placed on publication date; all relevant studies available up to October 2025 were considered to ensure comprehensive coverage of the topic. The search strategy employed Boolean operators (AND, OR) and was adapted to each database’s syntax. Keywords were grouped into six thematic domains to ensure broad coverage: BRD pathogenesis and host–pathogen interactions; Respiratory microbiome and virome; Diagnostic methodologies (including traditional and molecular approaches); Sequencing technologies (e.g., Illumina, Oxford Nanopore, NGS, metagenomics); Host defense mechanisms (URT/LRT, mucociliary clearance, innate immunity); Microbiome development and influencing factors (e.g., diet, stress, antimicrobials, vaccination).

Diagnostics were included as a core theme to reflect their central role in shaping BRD research—from early pathogen identification to the emergence of multi-omics approaches. The inclusion of both traditional and molecular diagnostics was intentional, as it illustrates the evolution of tools that underpin current understanding and surveillance strategies. Extracted studies were synthesized narratively and organized thematically. This approach enabled the identification of cross-cutting trends, methodological variability, and emerging knowledge gaps across BRD research domains. A total of 90 studies were included following the application of relevant filters.

## 3. Laboratory and Molecular Diagnostics for BRD

Diagnostic innovation is a central theme in the progression of BRD research, reflecting a shift from traditional pathogen detection methods to high-resolution, multi-omics approaches. Traditional diagnostics such as culture, ELISA, and immunohistochemistry (IHC) remain foundational in the diagnosis of BRD [22,23,24,25]. However, these approaches can be limited by factors such as low sensitivity, an inability to detect novel or unculturable pathogens, and a reliance on postmortem sampling [24,25,26].

### 3.1. Application of PCR Methodologies in the Diagnosis of BRD

Advances in molecular and proteomic technologies, such as multiplex real-time PCR and mass spectrometry, have enabled rapid, sensitive, and high-throughput detection and identification of BRD pathogens, surpassing traditional culture methods [27]. These innovations also allow for detailed sub-species typing, improving differentiation between pathogenic and commensal bacterial genotypes. PCR-based testing is used widely in veterinary diagnostics, and multiplex PCR assays now enable simultaneous detection of multiple pathogens, including mixed infections, with improved speed and specificity [4,5,28]. As multiple pathogens are often responsible for BRD onset this approach could be preferred over the more conventional PCR methodologies. Furthermore, the multiplexing of multiple targets saves on time and cost associated with the reaction. Multiplex qPCR approaches have been used to detect both bacterial and viral pathogens commonly associated with BRD [4,5,28,29,30,31]. This approach offers a useful tool for the rapid simultaneous detection of BRD pathogens.

Another PCR-based approach involves the use of loop-mediated isothermal amplification (LAMP) assays, which allow for the rapid amplification of pathogen-specific DNA under constant temperature conditions. LAMP assays offer rapid, field-deployable solutions for detecting key bacterial agents without the need for thermal cycling equipment [32,33,34]. These assays have been adapted for the detection of key BRD-associated bacteria, including *Mannheimia haemolytica*, *Pasteurella multocida*, and *Histophilus somni*, directly from nasal swab samples [32]. Although PCR-based methodologies have aided BRD diagnostics, a main disadvantage of PCR methodologies is the need for prior knowledge of the viral and bacterial genome sequences, which can lead to PCR bias in the results [35,36,37]. Furthermore, this targeted approach also means that unknown pathogens cannot be identified [38]. Other approaches, such as next generation sequencing (NGS) technologies do not require predesigned probes and so offer increased discovery power for unknown or novel genes/transcripts [34,35,36,37].

### 3.2. NGS Technologies for BRD Diagnosis

NGS refers to the extensive list of DNA sequencing technologies and applications that have revolutionized genomic-based research [39]. These platforms allow for unbiased profiling of host transcriptomes, bacterial microbiomes, and viral communities, enabling the discovery of novel pathogens and biomarkers [6,10,14,18]. A comparison of the laboratory and molecular tools available to support BRD diagnosis along with their strengths and limitations is detailed Table 1. Collectively, these innovations not only enhance diagnostic sensitivity and specificity but also support systems-level understanding of BRD pathogenesis. They enable the integration of host–pathogen–microbiome data, facilitate biomarker discovery, and inform targeted interventions and vaccine development.

## 4. Host Transcriptomics in BRD

RNA-Seq has become a popular choice in research and has the potential to identify biomarkers associated with human diseases, with the aim to improve disease diagnosis [40,41,42]. The same can be seen in the context of animal health with this approach used to examine the difference in gene expression in BRD versus healthy animals, in both naturally acquired disease as well as in response to experimental challenges with BRD-associated pathogens. An overview of studies that utilised this approach in the study of BRD are given in Table 2.

In addition to the study of mRNA, RNA-Seq can be used to examine non-coding RNAs. miRNAs are small (21–23 nucleotide) non-coding RNAs, that play a crucial role in the regulation of gene expression in plants and animals [49]. The comparison of miRNA profiles between different states (e.g., diseased versus healthy), allows expression patterns or specific miRNAs implicated in various biological processes or disease pathogenesis to be identified [50]. In humans, miRNAs have shown to have an association with a range of diseases [51], with studies suggesting miRNA-expression profiles have potential as diagnostic and prognostic biomarkers [52]. Similar has been shown in livestock [53], and it has been proposed that miRNAs play vital roles in the regulation of bovine immunity [54]. The association between miRNAs and antibody response of beef cattle to *Mycoplasma bovis (M. bovis)* was examined and four miRNAs showed a significant association to ELISA status, with these mainly involved in the host defence response to bacteria [55]. The miRNA expression profiles of bronchial lymph node tissue of dairy cattle following an experimental challenge with BRSV were examined, with 119 DE miRNAs identified in response to the viral challenge and genes targeted by these miRNAs were associated with pathogen recognition and interferon signalling [56]. Similar was conducted in beef cattle following experimental challenge with *M. bovis* or *M. bovis* and BVDV, whereby miRNA expression profiles of serum, white blood cells, liver, lymph nodes (mesenteric and trans-bronchial), spleen and thymus were examined [55]. Findings showed that certain miRNAs were higher in specific tissues compared to others and that differences in miRNA profiles were evident based on pathogen challenge [55]. Other studies conducted in vitro have examined the effect of BoHV-1 infection on miRNA expression with findings showing specific miRNAs to be involved in the suppression of BoHV-1 infection in MDBK cells [57].

Transcriptomic studies have significantly advanced our understanding of the bovine immune response to BRD, revealing consistent upregulation of genes involved in interferon signaling, pathogen recognition, and innate immunity across both natural and experimental infections. While whole blood transcriptomics offers a minimally invasive route for biomarker discovery, tissue-specific analyses, particularly of lymph nodes and lung, have uncovered localized gene expression patterns that may be critical for understanding pathogen tropism and disease progression. Notably, studies using experimental challenges with BoHV-1 and BRSV have provided clearer insights into host–pathogen interactions than those relying on naturally acquired BRD, where the initiating pathogen is often unknown. However, conflicting results in DEG profiles across studies suggest variability due to breed, sampling timepoints, and pathogen combinations. A major gap remains in the integration of transcriptomic data with other omics layers, such as miRNA and microbiome profiles, which could provide a more holistic view of BRD pathogenesis. Future research should prioritize longitudinal designs and multi-omics integration to identify robust biomarkers and therapeutic targets.

## 5. Characterization of the Bovine Respiratory Microbiome in BRD Infections

The term microbiome refers to the community of commensal, symbiotic and pathogenic microorganisms living within a particular environment [58]. In addition to the host’s structural and molecular defences, the microbiome of the bovine respiratory tract plays a key role in bovine health and disease [59]. The microbiome influences mucosal immunity, pathogen colonization resistance, and inflammatory responses. Disruptions in microbial balance—known as dysbiosis—can predispose cattle to infection by opportunistic pathogens and exacerbate disease progression. By characterizing microbial communities and their dynamics, researchers can identify microbial signatures associated with disease susceptibility, resilience, and progression. This knowledge supports the development of more integrated and predictive diagnostic tools, enabling earlier detection and targeted interventions for BRD.

### 5.1. Advances in BRD Diagnostics: Insights from 16S rRNA Gene Sequencing

NGS technologies have enabled the investigation of microbiomes in both humans and animals, with many studies utilising 16S ribosomal RNA (rRNA) gene sequencing to characterise bacterial microbiota [60]. Amplicon sequencing can be applied to almost all sample types [61], and to date has been used to study the microbiomes across a range of complex environments, with this approach able to identify both known and novel species. 16S rRNA amplicon sequencing uses a set of primers which, through PCR, bind to the conserved regions of the 16S rRNA gene [62]. A key benefit of this methodology is that it can be applied to samples of low biomass or those contaminated with host DNA [61].

In cattle, this approach has been used to identify BRD associated pathogens across the upper (URT) and lower (LRT) bovine respiratory tracts. An overview of the studies employing 16s rRNA sequencing in the study of BRD is provided in Table 3.

### 5.2. Third-Generation Sequencing Techniques in BRD Microbiome Characterisation

Genomes are complex and can have many long repeating elements and structural variations that are so long, short-read paired-end applications are sometimes unable to resolve [75,76]. To overcome this, long-read sequencing offering reads more than several kilobases, can span these complex regions with a single continuous read, eliminating ambiguity in the position or size of genomic elements [75]. Furthermore, third generation approaches offer simplified library preparation as well as the generation of real-time results [75]. Long-read sequencing (often dubbed third-generation sequencing) is available across a range of sequencing platforms. Today, one of the most widely adopted tools of the third-generation technologies is the MinION from ONT. As Nanopore sequencing does not require imaging equipment to detect nucleotides, it has allowed the system to be scaled down in size to a smaller, portable device, with the cost of these devices often far less compared to other sequencers [77]. Furthermore, the MinION device has potential for use in rapid field-based diagnostic laboratories [78]. The main drawback of long-read sequencing is the higher error rate compared to short read approaches [77].

Third-generation sequencing (MinION) was recently employed to identify BRD pathogens during natural BRD infection with findings showing an 89% concordance between this methodology and the traditional culture method [79]. More recently, a study characterized the bacterial microbiota of the upper and lower respiratory tracts in dairy calves following experimental infection with BoHV-1 [80]. In their study, calves were either inoculated with BoHV-1 or mock-challenged, and nasal swabs, pharyngeal tonsils, and lung tissues were collected for microbial analysis using ONT MinION sequencing. BoHV-1 successfully induced clinical BRD in challenged calves, validating the experimental model. At six days post-challenge, *Pasteurella*, *Streptococcus*, and *Ruminococcus* were the dominant genera identified across nasal, pharyngeal tonsil, and lung samples in infected calves. No significant differences in genus-level bacterial abundance were observed between challenged and control calves, although there was a trend toward altered Shannon diversity in pharyngeal tonsils, suggesting subtle shifts in microbial community structure. These findings indicate that BoHV-1 infection induces BRD without major alterations to the respiratory microbiota at the genus level within six days, while minor diversity changes may reflect early microbial responses to viral infection. The study also highlights the potential of MinION sequencing for rapid characterization of the bovine respiratory microbiome and its application in diagnostic settings.

Studies investigating the bovine respiratory microbiome have consistently shown that microbial diversity and composition are influenced by age, diet, stress, and disease status. BRD-affected cattle often exhibit reduced microbial richness and increased abundance of opportunistic pathogens such as *Mycoplasma*, *Pasteurella*, and *Histophilus* spp., particularly in the upper respiratory tract. However, findings vary across anatomical sites and study designs, with some studies reporting minimal changes in microbial composition following vaccination or antibiotic treatment. Notably, longitudinal studies have highlighted the dynamic nature of microbiomes in response to transportation and weaning stress, suggesting a window of vulnerability for BRD onset. Despite the widespread use of 16S rRNA gene sequencing, limitations in species-level resolution and the inability to detect functional interactions between microbial taxa persist. Emerging third-generation sequencing platforms, such as ONT MinION, offer improved resolution and field applicability, but require further validation. Overall, while the microbiome is clearly implicated in BRD susceptibility and progression, conflicting results and methodological variability highlight the need for standardized protocols and integrative multi-omics approaches to fully elucidate host–microbe–pathogen interactions.

## 6. Metagenomic Approaches to BRD Virome Characterization

In addition to the bacterial microbiota, the virome of the respiratory tract is also an area of interest. The application of viral metagenomics allows for the detection and characterization of known and novel viruses, offering a more complete understanding of the viral contributors to BRD.

The advent of metagenomic approaches has facilitated the characterization of the respiratory tract virome in bovines affected by BRD, revealing previously unrecognized viral agents [13,15,16,17,18,19,58,81]. In one of the earliest viral metagenomic studies, it was observed that cattle exhibiting clinical signs of BRD did not harbour the viruses traditionally associated with the disease, such as BoHV-1, BPI-3, and BRSV; instead, nucleic acid sequencing from nasopharyngeal swabs identified bovine rhinitis A virus (BRAV) and influenza D virus (IDV), suggesting the involvement of previously unrecognized viral agents [18].

Longitudinal sampling of nasopharyngeal swabs before transportation, on arrival at feedlots, and 40 days post-arrival demonstrated dynamic changes in the respiratory microbiome at all time points, which were attributed to stress and viral infection affecting both host immunity and microbiome composition [15]. Examination of the upper respiratory virome in nasal swabs from cattle in the USA and Mexico revealed geographical differences in viral composition, detecting BoHV-1, BVDV, BRSV, BCoV, IDV, BRV, REO, and BEV, highlighting a broader viral diversity than is currently addressed by vaccination [16].

Analysis of the respiratory virome in 130 post-slaughter beef cattle detected only a limited number of viruses, likely due to prior viral clearance by the host immune system and the preferential colonization of the upper respiratory tract, which reduces viral presence in the lungs. High amounts of non-viral nucleic acids, including host DNA contamination, were also found to reduce sequencing sensitivity [81]. Further studies analyzing deep nasal swabs and tracheal washes from both BRD-affected and clinically healthy cattle, all vaccinated against IBR, BVDV types I and II, BPIV-3, and BRSV, reported that these viruses were not detected; however, BoCV, BRBV, BRAV, and IDV were identified in diseased animals but not in healthy ones [82]. Nasal viromes of cattle on arrival at feedlots showed consistent patterns and further highlighted the role of BCoV, BRBV, BRAV, and IDV in BRD pathogenesis [17].

The use of high-throughput sequencing to characterize the bovine respiratory virome and its potential as a diagnostic tool was demonstrated using the ONT MinION device. When tested on in vitro viral cell cultures and nasal swabs from calves experimentally infected with a single BRD-associated DNA virus, BoHV-1, extensive optimization of Nanopore library preparation protocols, particularly to minimize PCR amplification bias, was required before BoHV-1 could be reliably detected as the dominant virus within approximately 7 h from sample to result [19].

Characterization of the upper respiratory tract virome of feedlot cattle in Australia confirmed associations between certain viral populations and BRD, highlighting the continued complexity and regional variation in viral contributors to the disease [13] Bovine coronavirus (BCoV) variants from nasal swabs of Irish calves with BRD, revealed genomic variation compared with previously reported strains [20]. In a subsequent study, they reported the genome of Ungulate tetraparvovirus 1 (UTPV1) from a nasal swab of a beef-suckler calf, representing one of the first characterizations of this virus in Ireland and contributing to knowledge of cattle virome diversity. Viruses including BRSV, BoHV-1, BVDV and PIV-3 are well documented as BRD opportunistic pathogens [3]. Previously, IDV had been isolated from nasal swabs of symptomatic BRD calves, which exhibited significantly higher levels of IDV RNA than those from healthy animals [16], and it was also shown to be significantly associated with BRD outcomes in Canada [81]. A diverse range of viruses were detected in the upper respiratory tract of Australian feedlot cattle. While some of these viruses are established causes of respiratory disease, the study highlights that numerous other viruses may also influence the development of BRD. Notably, there was a high abundance of bovine nidovirus (BoNV), IDV, BRAV, and BCoV across the samples, with BoNV being the most abundant RNA virus. Additionally, complete or near-complete genomes of BRBV, enterovirus E1, BVDV (sub-genotypes 1a and 1c), and BRSV were obtained, along with partial sequences of other viruses [21].

Like the bacterial microbiota, the bovine respiratory virome also appears to be a complex topic in BRD infection. Although some viruses appear to have an association with BRD, for others their role is not as clear. Furthermore, these identification of relatively newly associated BRD viruses, further highlights the importance of continued BRD diagnostic development that will aid in pathogen surveillance and identification in the future.

Metagenomic studies have expanded the understanding of the bovine respiratory virome, revealing a diverse array of viruses beyond classical BRD pathogens such as BoHV-1, BRSV, and BVDV. Emerging viruses—including bovine rhinitis A and B viruses (BRAV, BRBV), influenza D virus (IDV), and bovine nidovirus (BoNV)—have been increasingly detected in BRD-affected cattle, though their precise roles in disease pathogenesis remain unclear. Geographic variation and temporal dynamics further complicate interpretation, with some viruses appearing more prevalent in specific regions or at different stages of disease development. While high-throughput sequencing technologies, including ONT MinION, have enabled rapid and field-deployable viral detection, challenges persist in distinguishing pathogenic from commensal or opportunistic viruses. Conflicting findings, such as the presence of IDV in both healthy and diseased animals, underscore the need for longitudinal studies and functional validation. Overall, the virome represents a critical but underexplored component of BRD, and its integration with host transcriptomic and microbiome data will be essential to unravel complex host–virus–microbe interactions and improve diagnostic and preventive strategies.

## 7. Insights into BRD Pathogenesis

BRD pathogenesis typically begins with stress-induced immunosuppression, which compromises mucosal defences and facilitates colonization of the respiratory tract by viral and bacterial pathogens [1,3,12]. Primary viral infections—such as bovine herpesvirus 1 (BoHV-1), bovine respiratory syncytial virus (BRSV), and bovine viral diarrhoea virus (BVDV)—disrupt epithelial integrity and impair innate immune responses, creating a permissive environment for secondary bacterial invasion [3,10,11]. Bacterial pathogens including *Mannheimia haemolytica*, *Pasteurella multocida*, *Histophilus somni*, and *Mycoplasma bovis* contribute to disease progression through the production of virulence factors such as leukotoxins, adhesins, and endotoxins, which elicit inflammatory responses and tissue damage [28,30]. The resulting pulmonary lesions, characterized by fibrinous pneumonia and necrosis, are hallmarks of advanced BRD [3,14]. Recent advances in molecular diagnostics—such as metagenomics, RNA sequencing (RNA-Seq), and microRNA (miRNA) profiling—have enhanced our understanding of BRD pathogenesis [6,9,10,11,19,30]. These technologies enable the identification of novel pathogens, characterization of host transcriptomic responses, and elucidation of regulatory networks involved in immune modulation. Differential gene expression analyses have revealed activation of interferon signalling, complement pathways, and antimicrobial responses in infected tissues [6,7,8,10]. Additionally, miRNAs have been implicated in post-transcriptional regulation of immune genes, further refining the host response to infection [55,56]. Together, these insights support a systems-level understanding of BRD pathogenesis and inform the development of predictive biomarkers and targeted therapeutic strategies.

### Integrating Multi-Omics Approaches to Elucidate BRD Pathogenesis

Understanding BRD requires a systems-level perspective that captures the interplay between host immunity, pathogens, and the respiratory microbiome. Recent advances in transcriptomics, miRNA profiling, metagenomics, and microbiome characterization offer complementary insights, yet these datasets are often analyzed in isolation. Integrating these approaches can reveal mechanistic links between viral and bacterial colonization, host immune modulation, and microbial dysbiosis. Transcriptomic analyses have identified differentially expressed genes (DEGs) associated with innate immunity and interferon signalling in both whole blood and lymphoid tissues during BRD infection [10,11].

Similarly, miRNA studies highlight post-transcriptional regulation of antiviral pathways, with tissue-specific miRNA signatures linked to interferon signalling and pathogen recognition [56]. These host-level datasets provide a foundation for identifying biomarkers and immune pathways responsive to infection.

On the microbial side, 16S rRNA sequencing has demonstrated reduced species richness and increased abundance of opportunistic pathogens such as *Mycoplasma* and *Pasteurella* spp. in BRD-affected cattle [14,66]. Meanwhile, viral metagenomics has expanded the known virome, uncovering emerging viruses such as bovine rhinitis A virus (BRAV) and influenza D virus (IDV), which may act as primary or co-infecting agents [16,17,18].

**Figure 1:**
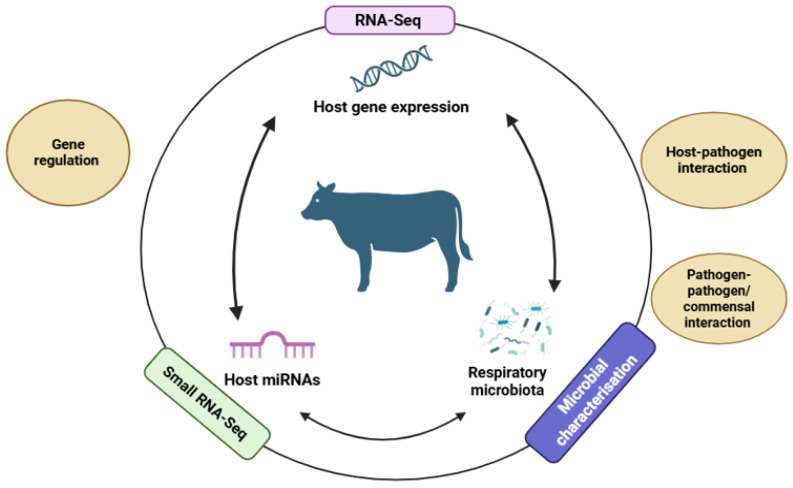
Conceptual model showing how multi-omics datasets could be combined to elucidate host–pathogen–microbiome interactions. Available online: https://app.biorender.com/illustrations/690892e8d877fbf4a3fc7066?slideId=6568a177-bdb9-4864-9990-40c4765f9c74 accessed on: 14 November 2025.

An integrative framework combining these layers—host transcriptome and miRNA profiles, pathogen detection via metagenomics, and microbiome composition—can elucidate host–pathogen–microbiome interactions driving BRD onset and progression. Figure 1 illustrates a conceptual model for multi-omics integration, highlighting key discovery targets and insights to be gained through the integration of these datasets.

## 8. Limitations in Current Omics Approaches for BRD Research

Despite significant advances in multi-omics technologies, several limitations constrain the interpretation and reproducibility of findings in BRD research.

Study Design and Sample Size: Many transcriptomic and microbiome studies employ small cohorts or convenience sampling, which restricts statistical power and limits generalizability of results. For example, RNA-Seq investigations of bronchial lymph nodes following experimental BRSV challenge included only 12 infected and 6 control calves [10], while microbiome studies comparing healthy and BRD-affected cattle often rely on single-site sampling [66]. These constraints underscore the need for longitudinal, multi-site designs to capture temporal dynamics and environmental variability.

Bioinformatics Variability: Differences in computational pipelines, normalization strategies, and reference databases can lead to inconsistent outcomes across studies. RNA-Seq workflows vary widely in alignment and differential expression analysis, influencing gene-level interpretations [83,84]. Similarly, microbiome profiling using 16S rRNA sequencing is sensitive to primer choice and taxonomic classifiers, which may bias community composition estimates.

Sequencing Depth and Contamination: Low sequencing depth can obscure rare taxa or low-abundance transcripts, reducing the resolution of microbial and virome analyses. Metagenomic studies of the bovine respiratory virome have reported challenges in detecting low-prevalence viruses and highlighted contamination risks from host DNA and environmental sources [81,85]. These technical limitations emphasize the importance of rigorous quality control, standardized protocols, and transparent reporting to ensure reproducibility and comparability of multi-omics datasets.

## 9. Conclusions

NGS technologies have allowed for the investigation into the impact of BRD on host animals at the molecular level. Approaches such as RNA-Seq have allowed for the examination of host gene expression in response to BRD infection [6,8,80,81], with genes and biological pathways involved in the host immune response to BRD identified. However, these studies analysed cattle with naturally acquired BRD, where the initial pathogen causing the infection is often unknown. The use of experimental challenge models utilising one or more specific BRD pathogens, coupled with these NGS approaches, offer deeper insight into the host immune response, allowing for the identification of DEGs in response to specific pathogens. Beyond mRNA, non-coding RNAs such as microRNAs (miRNAs), shown to play a role in immune function regulation [86], have also been analysed in cattle with experimentally induced BRD and have shown involvement in processes such as interferon signalling, pathogen recognition, and T-cell responses [56]. The examination of host responses using this approach enables the discovery of biomarkers of BRD infection, which may have potential for use in diagnostic development.

In addition to biomarker discovery, NGS technologies have been used to examine the significance of the URT [87,88] and LRT [89] respiratory tract microbiomes in respiratory tract health and disease susceptibility. Studies have analysed the respiratory microbiota of cattle with naturally occurring BRD [14,66,72], with findings showing certain species to play a role in respiratory health. However, the relative significance of different BRD pathogens remains unclear.

The application of NGS technologies in BRD research has allowed for the generation of many diverse datasets, facilitating the examination of the response to BRD at a deeper level. The integration of both gene expression, as well as microbial datasets provides the potential to further understand the cross-system response of BRD, not only at the host level, but also at a microbial level, through the characterisation of the microbiota residing within the hosts respiratory tract. The integration of these data will provide a deeper understanding of the impact of BRD on hosts themselves, as well as their respiratory microbiomes, which consequently may offer new insights into potential therapeutic targets.

The topics reviewed in this manuscript, including transcriptomics, metagenomics, microbiome dynamics, and molecular diagnostics, collectively support the stated objective by providing a comprehensive overview of the host–pathogen interactions and technological advances that have enhanced the understanding of BRD pathogenesis and diagnostic capabilities.

## 10. Future Directions and Research Priorities

BRD remains a multifactorial challenge requiring integrated diagnostic and management strategies. While significant progress has been made in applying transcriptomics, miRNA profiling, metagenomics, and microbiome characterization, these approaches are often siloed, limiting their translational impact. To enhance the impact of these approaches, future research should focus on the following priorities:

1. Standardization of Protocols:

There is a critical need to harmonize sampling methodologies, sequencing depth parameters, and bioinformatics pipelines. Standardization will reduce inter-study variability and improve reproducibility, thereby facilitating cross-comparative analyses and meta-analytical evaluations [83,84].

2. Multi-Omics Data Integration:

Developing robust frameworks for the integration of host transcriptomic and miRNA datasets with microbial and virome profiles is essential. Such integrative models should employ network-based analytics and machine learning techniques to elucidate host–pathogen–microbiome interactions, identify diagnostic biomarkers, and enable predictive modeling of disease outcomes [13,90].

3. Translational Applications:

To bridge the gap between research and field implementation, omics-derived insights must be translated into practical tools for BRD control. This includes the development of point-of-care diagnostic platforms utilizing portable sequencing technologies [10,11,19], as well as microbiome-informed interventions such as probiotics or targeted therapeutics [66].

By advancing these strategic priorities, the field will advance toward a systems-level understanding of BRD pathogenesis and enable evidence-based solutions for disease prevention and control.

## Figures and Tables

**Table 1:** BRD Diagnostics: A comparative overview.

Diagnostic Tool	Principle	Strengths	Weaknesses
**Culture**	Growth of pathogens on selective media	Gold standard for bacterial identification; inexpensive	Time-consuming; low sensitivity; cannot detect unculturable organisms
**ELISA**	Antigen–antibody interaction	High specificity; useful for herd-level screening	Requires trained personnel; limited multiplexing; cannot detect novel pathogens
**PCR (qPCR, multiplex)**	Amplification of target DNA/RNA	High sensitivity; rapid; multiplexing possible	Requires prior sequence knowledge; cannot detect unknown pathogens
**NGS (Illumina)**	High-throughput sequencing	Comprehensive pathogen profiling; detects novel species; high resolution	Expensive; requires bioinformatics expertise; longer turnaround
**Nanopore (MinION)**	Long-read sequencing via nanopores	Portable; real-time analysis; species-level resolution	Higher error rate than short-read; requires optimization for accuracy

**Table 2:** RNA-Seq-based studies examining the molecular immune response to BRD.

Animal Breed and Sample Size	Pathogen Challenge or Natural Infection	Tissue Investigated	Country	Key Findings	Reference
**Angus Hereford;** **Challenged (*n* = 4),** **control (*n* = 2)**	Pathogen challenge with one of the following (BRSV, BVDV, IBR, *M. haemolytica*, *P. multocida* or *M. bovis*)	Bronchial lymph node	USA	One hundred and forty-two differentially expressed genes were located in previously described quantitative trait locus regions associated with risk of BRD. DEGs were primarily involved in innate immunity pathways.	[9]
**Angus Hereford; Challenged (*n* = 4), control** **(*n* = 2)**	Pathogen challenge with one of the following (BRSV, BVDV, IBR, *M. haemolytica*, *P. multocida* or *M. bovis*)	Healthy and lesioned lung, bronchial lymph node, retropharyngeal lymph node, nasopharyngeal lymph node and pharyngeal tonsil	USA	Identified tissue specific transcriptional responses to the viral and bacterial pathogens. Identified gene networks involved in host innate immunity.	[43]
**Holstein-Friesian bull calves; BRSV (*n* = 12), control (*n* = 6)**	BRSV	Bronchial lymph node	Ireland	934 DEGs between BRSV and control calves. Enriched biological processes included interferon signalling, granzyme B signalling and pathogen pattern recognition receptors.	[10]
**Mixed breed Beef cattle (*n* = 24)**	Natural BRD infection	Blood	Canada	Gene expression profiles differed across Entry, Pulled and close out stages in each animal. The IFI6, IFIT3, ISG15, MX1 and OAS2 were identified as biomarkers to predict and recognize sick cattle.	[6]
**Beef cattle; BRD (*n* = 6), healthy controls (*n* = 5)**	Natural BRD infection	Blood	USA	132 DEGs identified between BRD and healthy cattle. Pathways related to microbial killing upregulated in cattle that contracted BRD.	[44]
**Mixed breed beef heifers; BRD (*n* = 25),** **Control (*n* = 18)**	Natural BRD infection	Blood	Canada	Identified a clear distinction in gene expression profiles between BRD and non-BRD cattle. Found similarities in DEGs with other studies such as *CATH2*, *LRG1*, *CFB*, *ALOX15* and *GZMB*.	[8]
**Beef cattle; BRD (*n* = 119), healthy (*n* = 115).**	Natural BRD infection	Blood	USA	Cattle diagnosed with BRD showed increase expression of genes associated with type I interferon production, alternative complement and granulocyte adhesion. Healthy cattle had increased expression of anti-inflammatory, antimicrobial and lymphatic maturation genes.	[7]
**Crossbred beef** **cattle;** **BRD mortality (*n* = 3),** **BRD survived (*n* = 3)**	Natural BRD infection	Blood	USA	Increase in expression of genes associated with pro-inflammation and immune response in cattle at arrival. Cattle that died from BRD show increased expression of type I interferon and antiviral genes at arrival.	[45]
**Holstein-Friesian bull calves; BRSV (*n* = 12), control (*n* = 6)**	BRSV challenge	Blood	Ireland	281 DEGs between BRSV and control calves. Enriched KEGG pathways were associated with viral infection including Influenza A, defense response to virus and innate immune response.	[46]
**Multi-breed beef cattle; BRD (*n* = 80), non-BRD** **(*n* = 63)**	Natural BRD infection	Blood	Canada	101 DEGs identified between BRD and non-BRD animals. *IL3RA* and *HBB* most significant upregulated and downregulated genes respectively.	[47]
**Cross-bred beef steers;** **(*n* = 43)**	Natural BRD infection	Blood	USA	Cattle that remained healthy had increased gene expression patterns relating to collagen formation and platelet activity compared to those that developed BRD.	[48]
**Holstein-Friesian bull calves, BoHV1 (*n* = 12), control (*n* = 6).**	BoHV-1 experimental challenge	Blood	Ireland		[11]

**Table 3:** An overview of studies investigating the bovine respiratory microbiome during BRD infection using a 16S rRNA sequencing approach is presented. For each study, the anatomical sampling site, study population, geographical location, and a summary of the key findings are provided. The table is organized into three sections: URT, LRT, and combined URT–LRT studies.

Anatomical Sampling Site(s)	Study Population	Geographical Location	Summary of the Key Findings	Reference
URT				
**Deep** **Nasopharyngeal** **swabs**	Holstein heifer calves (*n* = 174) in total. 37 diagnosed with pneumonia, 62 with otitis and 11 with pneumonia-otitis combined. 64 were healthy	USA	The relative abundance of *Mannheimia*, *Moraxella* and *Mycoplasma* were significantly higher in diseased versus healthy animals. Total bacterial load of newborn calves at day 3, was higher for animals that developed pneumonia compared to those that remained healthy.	[63]
**Deep** **Nasopharyngeal** **swabs**	BRD (*n* = 22) and pen matched healthy controls (*n* = 10).	USA	The overall composition of the BRD calves was different from that of the healthy controls. Predominant genera were Moraxella, Mycoplasma and Acinetobacter Nasopharyngeal microbiota differed in feedlot calves at entry and in BRD calves, compared to healthy controls.	[64]
**Nasopharyngeal** **swabs**	Crossbred beef steer calves (*n* = 120). Three groups; Spring processing, at arrival and 40 days after arrival (*n* = 40 calves per group)	Canada	Mycoplasma was the most abundant genus and *M. dispar* the most abundant species across all groups. Difference in the composition of the microbiota over time for all calf groups.	[65]
**Nasopharyngeal** **swabs**	Feedlot cattle with (*n* = 82) and without BRD (*n* = 82)	Canada	Species richness was lower in BRD cattle compared to controls. Health status and days on feed were sources of variation for microbiota composition. *M. bovis* was more frequently identified in cattle with BRD.	[66]
**Nasal cavity**	Holstein steers (6–7 months old) (*n* = 75 healthy; *n =* 58 BRD)	USA	BRD cattle had lower alpha diversity compared to controls. *Trueperella pyogenes*, *Bibersteinia* and *Mycoplasma* spp. were increased in relative abundance in the BRD group, while *Mycoplasma bovirhinis* and *Clostridium sensu stricto* were increased in the healthy group. The prevalence of *H. somni* and *P. multocida* were high regardless of clinical status. *M. haemolytica* and *M. bovis* were more prevalent in the BRD group.	[67]
**Nasal swabs**	Feedlot calves (*n* = 51)	USA	Neither bovine coronavirus nor *Mycoplasma* sp. were present at high abundance at the earlier timepoint of initial vaccination. Alpha diversity was significantly greater at initial vaccination compared to the BRD outbreak (*p*-value < 0.001). At the time of the BRD outbreak, all calves were nasally shedding bovine coronavirus and a large percentage had a coinfection with *Mycoplasma* sp., with *Mycoplasma bovirhinis* being the predominant species.	[68]
**Nasopharynx (nasal swabs)**	Crossbred beef-bull and steer calves. Two treatment groups (vaccines) INT (*n* = 175) and INJ (*n* = 175). Control group (*n* = 175)	USA	The microbiome in healthy animals on d 28 had increased Proteobacteria (largely *Moraxella* spp.) and decreased Firmicutes (comprising almost exclusively of *Mycoplasma* spp.) compared to animals that were treated for or died from BRD (*p* < 0.05). A greater RA of *Mycoplasma* spp. was observed in cattle that died of BRD on d0. All animals displayed an increased diversity on d 28.	[69]
** LRT **				
**Lung and mediastinal lymph node**	Cranial lung lobe from beef (*n*= 32) and dairy (*n* = 6) calves. Mediastinal lymph node from beef calves (*n* = 32)	Ireland	*Leptotrichiaceae*, *Mycoplasma*, *Pasteurellaceae*, and *Fusobacterium* were the most abundant OTUs identified in the lungs and lymph nodes of the calves which died from BRD. Certain bacterial genera had greater relative abundance in the post-mortem lung samples collected from dairy calves that died from BRD compared to healthy controls with no lesions present. *Leptotrichiaceae* OTUs were sequenced and found not to be identical to any known bacterial genus, suggesting the identification of a novel bacterial species associated with BRD.	[14]
**Cranial lung lobe**	Feedlot calves with BRD (*n* = 6) and clinically healthy controls (*n* = 6)	Egypt	Statistically significant variations in abundance at the family and genus levels. Statistically significant differences in chao1 and Shannon diversity observed between the two groups. Beta diversity analysis displayed a clear difference (*p* = 0.044) between the microbiota of healthy versus BRD calves. A core microbiota of 188 OTUs was found to be shared between the two groups.	[70]
** URT– LRT **				
**Nasal swabs and trans-tracheal aspirations (TTA)**	Piedmontese calves with (*n* = 8) and without (*n* = 11) clinical signs of respiratory disease	Italy	Twenty-nine phyla and 305 genera were identified. Mycoplasma was the most abundant genus in the nasal and TTA samples. *Pasteurella multocida* and *Psychrobacter sanguinis* were the most abundant species in the nasal and TTA samples, respectively. No significant difference was observed between the nasal and TTA samples based on clinical signs. NS differed by farm origin when compared using unweighted UniFrac metric (*p* = 0.05).	[71]
**Nasal cavity and Bronchoalveolar lavage**	Charolais calves (*n* = 8)	USA		[64]
**Nasopharyngeal swabs and tracheal washes**	Recently weaned crossbred beef-breed heifer calves (*n* = 24)	Canada	No common pattern of change observed in nasopharyngeal or tracheal microbiotas. Variation among animals and time affected microbiota more than health status. Moraxella and Mycoplasma suggested to play a role in respiratory health.	[72]
**Nostrils, nasopharynx, oropharynx, hard palate, floor of mouth, palatine tonsils, trachea, bronchus, bronchi**	Crossbred beef-breed feedlot steer calves (*n* = 18)	Canada	Bacterial communities varied dependent on anatomical location. Nasopharyngeal flora was most similar to that of the lung bacterial microbiome.	[73]
**Nasopharynx, trachea, lung and joint**	Feedlot cattle that died from BRD (*n* = 32) and those that died of other causes (control) (*n* = 8)	Canada	Lower bacterial diversity in the nasopharynx, trachea and lungs of cattle that died from BRD compared to other causes. In cattle that died of BRD, alpha-diversity was lower in the lungs and joints compared to the nasopharynx. The relative abundance of *Mycoplasma* spp. in the lung, *Pasteurella* spp. in the trachea and lung, and *Histophilus* spp. in the lung, trachea and nasopharynx of cases were higher (*p* < 0.001) than controls. Cattle that died of BRD had less diverse respiratory microbiomes with a higher abundance of respiratory pathogens.	[74]
**Nasal swabs**	Holstein and Jersey calves and cows. BRD (*n* = 50) and healthy (*n* = 50)	USA	The genera *Acinetobacter*, *Moraxella*, *Psychrobacter*, *Histophilus*, *Mannheimia*, *Mycoplasma*, and *Pasteurella* were prevalent in the bovine nasal microbiome regardless of farm or disease status. *H. somni* was most prevalent whilst *M. bovis* was least prevalent. At one farm location (CA), the abundance of a pathobiont differed according to disease status, where *M. haemolytica* was significantly more abundant in the BRD-affected animals than apparently healthy animals.	[67]

## Data Availability

No new data were created or analyzed in this study.

## Abbreviations

The following abbreviations are used in this paper:

BCoV	Bovine coronavirus
BoHV-1	Bovine herpesvirus 1
BPI3V	Bovine parainfluenza 3
BRAV1	Bovine rhinitis A virus 1
BRAV2	Bovine rhinitis A virus 2
BRD	Bovine respiratory disease
BRSV	Bovine respiratory syncytial virus
BVDV	Bovine viral diarrhoea virus
cDNA	Complementary DNA)
LAMP	Loop-mediated isothermal amplification
LASL	Linker Amplified Shotgun Library
LRT	Lower respiratory tract
MDA	Multiple Displacement Amplification
MLN	Mediastinal lymph node
MiRNA	MicroRNA
MLV	Modified live vaccine
mRNA	Messenger RNA
NCBI	National Center for Biotechnology Information
OTUs	Operational Taxonomic Units
RNA	Ribonucleic acid
RNA-Seq	RNA-Sequencing
SISPA	Sequence-Independent Single-Primer Amplification
URT	Upper respiratory tract
US	United States

## References

[1] Pardon B., Buczinski S. (2020). Bovine respiratory disease diagnosis: What progress has been made in infectious diagnosis?. Vet. Clin. North Am. Food Anim. Pract..

[2] Kamel M.S., Davidson J.L., Verma M.S. (2024). Strategies for bovine respiratory disease (BRD) diagnosis and prognosis: A comprehensive overview. Animals.

[3] O’Donoghue S., Waters S.M., Morris D.W., Earley B. (2025). A comprehensive review: Bovine respiratory disease, current insights into epidemiology, diagnostic challenges, and vaccination. Vet. Sci..

[4] Thonur L., Maley M., Gilray J., Crook T., Laming E., Turnbull D., Nath M., Willoughby K. (2012). One-step multiplex real-time RT-PCR for the detection of bovine respiratory syncytial virus, bovine herpesvirus 1, and bovine parainfluenza virus 3. BMC Vet. Res..

[5] Hao F., Tao C., Xiao R., Huang Y., Yuan W., Wang Z., Jia H. (2025). Development of a Multiplex Real-Time PCR Assay for the detection of eight pathogens associated with bovine respiratory disease complex from clinical samples. Microorganisms.

[6] Sun H.Z., Srithayakumar V., Jiminez J., Jin W., Hosseini A., Raszek M., Orsel K., Guan L.L., Plastow G. (2020). Longitudinal blood transcriptomic analysis to identify molecular regulatory patterns of bovine respiratory disease in beef cattle. Genomics.

[7] Scott M.A., Woolums A.R., Swiderski C.E., Finley A., Perkins A.D., Nanduri B., Karisch B.B. (2022). Hematological and gene co-expression network analyses of high-risk beef cattle defines immunological mechanisms and biological complexes involved in bovine respiratory disease and weight gain. PLoS ONE.

[8] Jiminez J., Timsit E., Orsel K., van der Meer F., Guan L.L., Plastow G. (2021). Whole-blood transcriptome analysis of feedlot cattle with and without bovine respiratory disease. Front. Genet..

[9] Tizioto P.C., Kim J., Seabury C.M., Schnabel R.D., Gershwin L.J., Van Eenennaam A.L., Toaff-Rosenstein R., Neibergs H.L., Team B.R.D.C.C.A.P.R., Taylor J.F. (2015). Immunological response to single pathogen challenge with agents of the bovine respiratory disease complex: An RNA-sequence analysis of the bronchial lymph node transcriptome. PLoS ONE.

[10] Johnston D., Earley B., McCabe M.S., Lemon K., Duffy C., McMenamy M., Cosby S.L., Kim J., Blackshields G., Taylor J.F. (2019). Experimental challenge with bovine respiratory syncytial virus in dairy calves: Bronchial lymph node transcriptome response. Sci. Rep..

[11] O’Donoghue S., Earley B., Johnston D., McCabe M.S., Kim J.W., Taylor J.F., Duffy C., Lemon K., McMenamy M., Cosby S.L. (2023). Whole blood transcriptome analysis in dairy calves experimentally challenged with bovine herpesvirus 1 (BoHV-1) and comparison to a bovine respiratory syncytial virus (BRSV) Challenge. Front. Genet..

[12] Murray G.M., O’Neill R.G., More S.J., McElroy M.C., Earley B., Cassidy J.P. (2016). Evolving views on bovine respiratory disease: An appraisal of selected key pathogens–Part 1. Vet. J..

[13] Ambrose R.K., Blakebrough-Hall C., Gravel J.L., Gonzalez L.A., Mahony T.J. (2023). Characterisation of the upper respiratory tract virome of feedlot cattle and its association with bovine respiratory disease. Viruses.

[14] Johnston D., Earley B., Cormican P., Murray G., Kenny D.A., Waters S.M., McGee M., Kelly A.K., McCabe M.S. (2017). Illumina MiSeq 16S Amplicon sequence analysis of bovine respiratory disease associated bacteria in lung and mediastinal lymph node tissue. BMC Vet. Res..

[15] Timsit E., Workentine M., Schryvers A.B., Holman D.B., van der Meer F., Alexander T.W. (2016). Evolution of the nasopharyngeal microbiota of beef cattle from weaning to 40 days after arrival at a feedlot. Vet. Microbiol..

[16] Mitra N., Cernicchiaro N., Torres S., Li F., Hause B.M. (2016). Metagenomic Characterization of the virome associated with bovine respiratory disease in feedlot cattle identified novel viruses and suggests an etiologic role for influenza D virus. J. Gen. Virol..

[17] Zhang M., Hill J.E., Alexander T.W., Huang Y. (2021). The nasal viromes of cattle on arrival at western Canadian feedlots and their relationship to development of bovine respiratory disease. Transbound. Emerg. Dis..

[18] Ng T.F.F., Kondov N.O., Deng X., Van Eenennaam A., Neibergs H.L., Delwart E. (2015). A Metagenomics and case-control study to identify viruses associated with bovine respiratory disease. J. Virol..

[19] Esnault G., Earley B., Cormican P., Waters S.M., Lemon K., Cosby S.L., Lagan P., Barry T., Reddington K., McCabe M.S. (2022). Assessment of Rapid MinION nanopore DNA virus metagenomics using calves experimentally infected with bovine herpes virus-1. Viruses.

[20] Ní Dhufaigh K., McCabe M., Cormican P., Cuevas-Gomez I., McGee M., McDaneld T., Earley B. (2022). Genome sequence of bovine coronavirus variants from the nasal virome of Irish beef suckler and pre-weaned dairy calves clinically diagnosed with bovine respiratory disease. Microbiol. Resour. Announc..

[21] Brito B.P., Frost M.J., Anantanawat K., Jaya F., Batterham T., Djordjevic S.P., Chang W.S., Holmes E.C., Darling A.E., Kirkland P.D. (2023). Expanding the range of the respiratory infectome in australian feedlot cattle with and without respiratory disease using metatranscriptomics. Microbiome.

[22] Gershwin L.J., Van Eenennaam A.L., Anderson M.L., McEligot H.A., Shao M.X., Toaff-Rosenstein R., Taylor J.F., Neibergs H.L., Womack J., Bovine Respiratory Disease Complex Coordinated Agricultural Project Research Team (2015). Single pathogen challenge with agents of the bovine respiratory disease complex. PLoS ONE.

[23] Poonsuk K., Kordik C., Hille M., Cheng T.-Y., Crosby W.B., Woolums A.R., Clawson M.L., Chitko-McKown C., Brodersen B., Loy J.D. (2023). Detection of Mannheimia haemolytica-specific IgG, IgM and IgA in sera and their relationship to respiratory disease in cattle. Animals.

[24] Werid G.M., Miller D., Hemmatzadeh F., Messele Y.E., Petrovski K. (2023). An overview of the detection of bovine respiratory disease complex pathogens using immunohistochemistry: Emerging trends and opportunities. J. Vet. Diagn. Investig..

[25] Singh S., Singh R., Singh K.P., Singh V., Malik Y.P.S., Kamdi B., Singh R., Kashyap G. (2020). Immunohistochemical and molecular detection of natural cases of bovine rotavirus and coronavirus infection causing enteritis in dairy calves. Microb. Pathog..

[26] Fulton R.W., Confer A.W. (2012). Laboratory test descriptions for bovine respiratory disease diagnosis and their strengths and weaknesses: Gold standards for diagnosis, do they exist?. Can. Vet. J..

[27] Loy J.D. (2020). Development and application of molecular diagnostics and proteomics to bovine respiratory disease (BRD). Anim. Health Res. Rev..

[28] Kishimoto M., Tsuchiaka S., Rahpaya S.S., Hasebe A., Otsu K., Sugimura S., Kobayashi S., Komatsu N., Nagai M., Omatsu T. (2017). Development of a One-Run Real-Time PCR detection system for pathogens associated with bovine respiratory disease complex. J. Vet. Med. Sci..

[29] Wisselink H.J., Cornelissen J.B., van der Wal F.J., Kooi E.A., Koene M.G., Bossers A., Smid B., de Bree F.M., Antonis A.F. (2017). Evaluation of a Multiplex Real-Time PCR for detection of four bacterial agents commonly associated with bovine respiratory disease in bronchoalveolar lavage fluid. BMC Vet. Res..

[30] Goto Y., Yaegashi G., Fukunari K., Suzuki T. (2020). Design of a Multiplex Quantitative Reverse Transcription-PCR system to simultaneously detect 16 pathogens associated with bovine respiratory and enteric diseases. J. Appl. Microbiol..

[31] Zhang J., Wang W., Yang M., Lin J., Xue F., Zhu Y., Yin X. (2022). Development of a one-step multiplex real-time PCR assay for the detection of viral pathogens associated with the bovine respiratory disease complex. Front. Vet. Sci..

[32] Pascual-Garrigos A., Maruthamuthu M.K., Ault A., Davidson J.L., Rudakov G., Pillai D., Koziol J., Schoonmaker J.P., Johnson T., Verma M.S. (2021). On-farm colorimetric detection of Pasteurella multocida, Mannheimia haemolytica, and Histophilus somni in Crude Bovine Nasal Samples. Vet. Res..

[33] Mohan S., Pascual-Garrigos A., Brouwer H., Pillai D., Koziol J., Ault A., Schoonmaker J., Johnson T., Verma M.S. (2021). Loop-Mediated Isothermal Amplification for the detection of Pasteurella multocida, Mannheimia haemolytica, and Histophilus somni in Bovine Nasal Samples. ACS Agric. Sci. Technol..

[34] Petrini S., Iscaro C., Righi C. (2019). Antibody responses to bovine alphaherpesvirus 1 (BoHV-1) in passively immunized calves. Viruses.

[35] Zhang M., Huang Y., Godson D.L., Fernando C., Alexander T.W., Hill J.E. (2020). Assessment of metagenomic sequencing and qPCR for detection of influenza D virus in bovine respiratory tract samples. Viruses.

[36] Pinto A.J., Raskin L. (2012). PCR biases distort bacterial and archaeal community structure in pyrosequencing datasets. PLoS ONE.

[37] Silverman J.D., Bloom R.J., Jiang S., Durand H.K., Dallow E., Mukherjee S., David L.A. (2021). Measuring and mitigating PCR bias in microbiota datasets. PLoS Comput. Biol..

[38] Kralik P., Ricchi M. (2017). A Basic Guide to Real-Time PCR in Microbial Diagnostics: Definitions, parameters, and everything. Front. Microbiol..

[39] Behjati S., Tarpey P.S. (2013). What is next generation sequencing?. Arch. Dis. Child. Educ. Pract. Ed..

[40] Iqbal N., Kumar P. (2022). Integrated COVID-19 Predictor: Differential expression analysis to reveal potential biomarkers and prediction of Coronavirus Using RNA-Seq Profile Data. Comput. Biol. Med..

[41] Wei I.H., Shi Y., Jiang H., Kumar-Sinha C., Chinnaiyan A.M. (2014). RNA-Seq accurately identifies cancer biomarker signatures to distinguish tissue of origin. Neoplasia.

[42] Li J., Wang H., Mao L., Yu H., Yu X., Sun Z., Qian X., Cheng S., Chen S., Chen J. (2020). Rapid genomic characterization of SARS-CoV-2 viruses from clinical specimens using nanopore sequencing. Sci. Rep..

[43] Behura S.K., Tizioto P.C., Kim J., Grupioni N.V., Seabury C.M., Schnabel R.D., Gershwin L.J., Van Eenennaam A.L., Toaff-Rosenstein R., Neibergs H.L. (2017). Tissue tropism in host transcriptional response to members of the bovine respiratory disease complex. Sci. Rep..

[44] Scott M.A., Woolums A.R., Swiderski C.E., Perkins A.D., Nanduri B., Smith D.R., Karisch B.B., Epperson W.B., Blanton J.R. (2020). Whole blood transcriptomic analysis of beef cattle at arrival identifies potential predictive molecules and mechanisms that indicate animals that naturally resist bovine respiratory disease. PLoS ONE.

[45] Scott M.A., Woolums A.R., Swiderski C.E., Perkins A.D., Nanduri B., Smith D.R., Karisch B.B., Epperson W.B., Blanton J.R. (2021). Comprehensive at-arrival transcriptomic analysis of post-weaned beef cattle uncovers type I interferon and antiviral mechanisms associated with bovine respiratory disease mortality. PLoS ONE.

[46] Johnston D., Earley B., McCabe M.S., Kim J., Taylor J.F., Lemon K., Duffy C., McMenamy M., Cosby S.L., Waters S.M. (2021). Messenger RNA biomarkers of bovine respiratory syncytial virus infection in the whole blood of dairy calves. Sci. Rep..

[47] Li Z., Li X., Jin M., Liu Y., He Y., Jia N., Cui X., Liu Y., Hu G., Yu Q. (2022). Identification of potential biomarkers for early diagnosis of schizophrenia through RNA sequencing analysis. J. Psychiatr. Res..

[48] Green M.M., Woolums A.R., Karisch B.B., Harvey K.M., Capik S.F., Scott M.A. (2023). Influence of the at-arrival host transcriptome on bovine respiratory disease incidence during backgrounding. Vet. Sci..

[49] Glazov E.A., Kongsuwan K., Assavalapsakul W., Horwood P.F., Mitter N., Mahony T.J. (2009). Repertoire of bovine mirna and mirna-like small regulatory rnas expressed upon viral infection. PLoS ONE.

[50] Tam S., Tsao M.-S., McPherson J.D. (2015). Optimization of miRNA-seq data preprocessing. Brief. Bioinform..

[51] Wang J., Chen J., Sen S. (2016). MicroRNA as biomarkers and diagnostics. J. Cell. Physiol..

[52] Huang W. (2017). MicroRNAs: Biomarkers, Diagnostics, and Therapeutics. Bioinformatics in MicroRNA Research.

[53] Miretti S., Lecchi C., Ceciliani F., Baratta M. (2020). MicroRNAs as Biomarkers for animal health and welfare in livestock. Front. Vet. Sci..

[54] Dong H., Gao Q., Peng X., Sun Y., Han T., Zhao B., Liu Y., Wang C., Song X., Wu J. (2017). Circulating MicroRNAs as potential biomarkers for veterinary infectious diseases. Front. Vet. Sci..

[55] Casas E., Cai G., Kuehn L.A., Register K.B., McDaneld T.G., Neill J.D. (2016). Association of MicroRNAs with antibody response to Mycoplasma bovis in beef cattle. PLoS ONE.

[56] Johnston D., Earley B., McCabe M.S., Kim J., Taylor J.F., Lemon K., McMenamy M., Duffy C., Cosby S.L., Waters S.M. (2021). Elucidation of the host bronchial lymph node miRNA transcriptome response to bovine respiratory syncytial virus. Front. Genet..

[57] Hou P., Zhao M., He W., He H., Wang H. (2019). Cellular MicroRNA bta-miR-2361 Inhibits Bovine Herpesvirus 1 replication by directly targeting EGR1 Gene. Vet. Microbiol..

[58] Lederberg J., McCray A.T. (2001). Ome Sweet Omics—A genealogical treasury of words. Scientist.

[59] Zeineldin M., Lowe J., Aldridge B. (2019). Contribution of the mucosal microbiota to bovine respiratory health. Trends Microbiol..

[60] Fouhy F., Clooney A.G., Stanton C., Claesson M.J., Cotter P.D. (2016). 16S rRNA Gene Sequencing of mock microbial populations—Impact of DNA extraction method, primer choice and sequencing platform. BMC Microbiol..

[61] Liu L., Li Y., Li S., Hu N., He Y., Pong R., Lin D., Lu L., Law M. (2012). Comparison of next-generation sequencing systems. Biomed. Res. Int..

[62] Klindworth A., Pruesse E., Schweer T., Peplies J., Quast C., Horn M., Glöckner F.O. (2013). Evaluation of general 16S ribosomal RNA Gene PCR primers for classical and next-generation sequencing-based diversity studies. Nucleic Acids Res..

[63] Lima S.F., Teixeira A.G., Higgins C.H., Lima F.S., Bicalho R.C. (2016). The Upper Respiratory tract microbiome and its potential role in bovine respiratory disease and otitis media. Sci. Rep..

[64] Zeineldin M., Lowe J., de Godoy M., Maradiaga N., Ramirez C., Ghanem M., Abd El-Raof Y., Aldridge B. (2017). Disparity in the nasopharyngeal microbiota between healthy cattle on feed, at entry processing, and with respiratory disease. Vet. Microbiol..

[65] McMullen C., Orsel K., Alexander T.W., van der Meer F., Plastow G., Timsit E. (2018). Evolution of the nasopharyngeal bacterial microbiota of beef calves from spring processing to 40 days after feedlot arrival. Vet. Microbiol..

[66] McMullen C., Orsel K., Alexander T.W., van der Meer F., Plastow G., Timsit E. (2019). Comparison of the nasopharyngeal bacterial microbiota of beef calves raised without the use of antimicrobials between healthy calves and those diagnosed with bovine respiratory disease. Vet. Microbiol..

[67] Centeno-Delphia R.E., Glidden N., Long E., Ellis A., Hoffman S., Mosier K., Ulloa N., Cheng J.J., Davidson J.L., Mohan S. (2025). Nasal pathobiont abundance is a moderate feedlot-dependent indicator of bovine respiratory disease in beef cattle. Anim. Microbiome.

[68] McDaneld T.G., Workman A.M., Chitko-McKown C.G., Kuehn L.A., Dickey A., Bennett G.L. (2022). Detection of Mycoplasma bovirhinis and Bovine Coronavirus in an outbreak of bovine respiratory disease in nursing beef calves. Front. Microbiomes.

[69] McAtee T.B., Pinnell L.J., Powledge S.A., Wolfe C.A., Morley P.S., Richeson J.T. (2023). Effects of respiratory virus vaccination and bovine respiratory disease on the respiratory microbiome of feedlot cattle. Front. Microbiol..

[70] Sabry I., Zeineldin M., Kamal M., Hefnawy A., El-Attar H., Abdelraof Y., Ghanem M. (2025). Comparative evaluation of lower respiratory tract microbiota in healthy and BRD-affected calves in Egypt. Trop. Anim. Health Prod..

[71] Nicola I., Cerutti F., Grego E., Bertone I., Gianella P., D’Angelo A., Peletto S. (2017). Characterization of the upper and lower respiratory tract microbiota in piedmontese Calves. Microbiome.

[72] McMullen C., Alexander T.W., Léguillette R., Workentine M., Timsit E. (2020). Topography of the Respiratory Tract Bacterial Microbiota in Cattle. Microbiome.

[73] McMullen C., Alexander T.W., Orsel K., Timsit E. (2020). Progression of Nasopharyngeal and tracheal bacterial microbiotas of feedlot cattle during development of bovine respiratory disease. Vet. Microbiol..

[74] Li C., Zaheer R., Kinnear A., Jelinski M., McAllister T.A. (2022). Comparative microbiomes of the respiratory tract and joints of feedlot cattle mortalities. Microorganisms.

[75] Goodwin S., McPherson J.D., McCombie W.R. (2016). Coming of Age: Ten Years of Next-Generation Sequencing Technologies. Nat. Rev. Genet..

[76] Xiao T., Zhou W. (2020). The third generation sequencing: The advanced approach to genetic diseases. Transl. Pediatr..

[77] Kono N., Arakawa K. (2019). Nanopore Sequencing: Review of potential applications in functional genomics. Dev. Growth Differ..

[78] Walter M.C., Zwirglmaier K., Vette P., Holowachuk S.A., Stoecker K., Genzel G.H., Antwerpen M.H. (2017). MinION as part of a biomedical rapidly deployable laboratory. J. Biotechnol..

[79] Okamura S., Fukuda A., Usui M. (2024). Rapid detection of causative bacteria including multiple infections of bovine respiratory disease using 16S rRNA amplicon-based nanopore sequencing. Vet. Res. Commun..

[80] O’Donoghue S., Earley B., McCabe M.S., Johnston D., Cosby S.L., Lemon K., Kim J., Taylor J.F., Morris D., Waters S. (2025). Characterisation of the bacterial microbiota of nasal swab and pharyngeal tonsil samples from dairy calves following experimental challenge with bovine herpesvirus 1 (BoHV-1). Anim.–Sci. Proc..

[81] Zhang M., Hill J.E., Fernando C., Alexander T.W., Timsit E., van der Meer F., Huang Y. (2019). Respiratory viruses identified in western Canadian beef cattle by metagenomic sequencing and their association with bovine respiratory disease. Transbound. Emerg. Dis..

[82] Zhang M., Hill J.E., Godson D.L., Ngeleka M., Fernando C., Huang Y. (2020). The pulmonary virome, bacteriological and histopathological findings in bovine respiratory disease from western Canada. Transbound. Emerg. Dis..

[83] Costa-Silva J., Domingues D., Lopes F.M. (2017). RNA-Seq Differential Expression Analysis: An Extended Review and a Software Tool. PLoS ONE.

[84] Deshpande D., Chhugani K., Chang Y., Karlsberg A., Loeffler C., Zhang J., Muszyńska A., Munteanu V., Yang H., Rotman J. (2023). RNA-seq Data science: From raw data to effective interpretation. Front. Genet..

[85] Medina J.E., Castañeda S., Camargo M., García-Corredor D.J., Muñoz M., Ramírez J.D. (2024). Exploring viral diversity and metagenomics in livestock: Insights into disease emergence and spillover risks in cattle. Vet. Res. Commun..

[86] Sonkoly E., Ståhle M., Pivarcsi A. (2008). MicroRNAs and immunity: Novel players in the regulation of normal immune function and inflammation. Semin. Cancer Biol..

[87] Holman D.B., McAllister T.A., Topp E., Wright A.-D.G., Alexander T.W. (2015). The Nasopharyngeal microbiota of feedlot cattle that develop bovine respiratory disease. Vet. Microbiol..

[88] McDaneld T.G., Kuehn L.A., Keele J.W. (2018). Evaluating the Microbiome of Two Sampling Locations in the Nasal Cavity of Cattle with Bovine Respiratory Disease Complex (BRDC)1. J. Anim. Sci..

[89] Klima C.L., Holman D.B., Ralston B.J., Stanford K., Zaheer R., Alexander T.W., McAllister T.A. (2019). Lower respiratory tract microbiome and resistome of bovine respiratory disease mortalities. Microb. Ecol..

[90] Qi J., Huang F., Gan L., Zhou X., Gou L., Xie Y., Guo H., Fang J., Zuo Z. (2024). Multi-omics investigation into long-distance road transportation effects on respiratory health and immunometabolic responses in calves. Microbiome.

